# Supplementary figures and images for: Community purchases of antimicrobials during the COVID-19 pandemic in Uganda: An increased risk for antimicrobial resistance
Source: PLOS Glob Public Health. 2023 Feb 23;3(2):e0001579. doi: 10.1371/journal.pgph.0001579 (PMC10021632; doi:10.1371/journal.pgph.0001579)

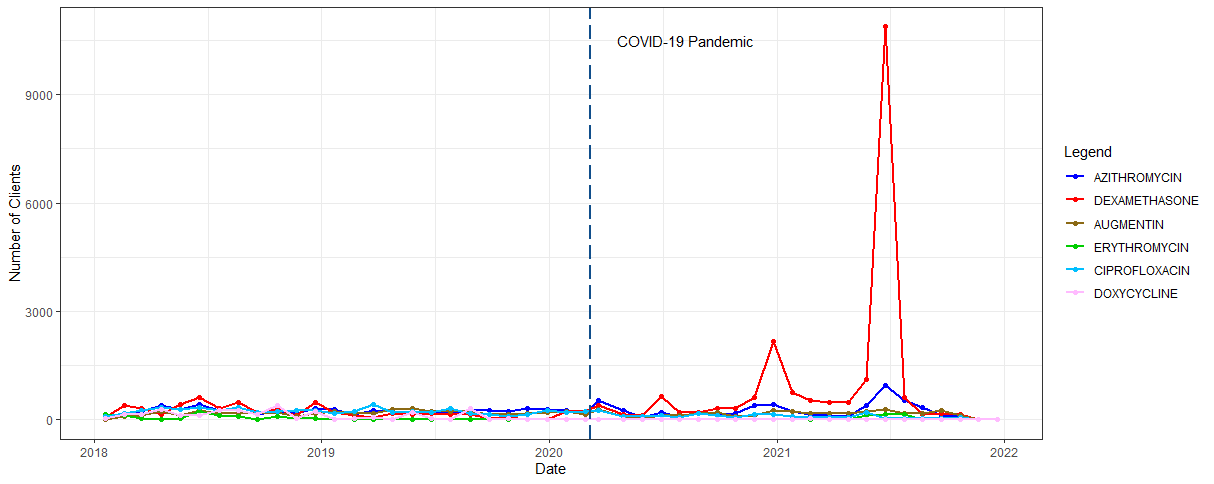

Supplement: S1 Fig — The time points correspond to the months and years before and during COVID-19. For example, March 2020 corresponds to the month in which the first COVID-19 case was diagnosed in Uganda. (TIFF) [file pgph.0001579.s001.tiff]

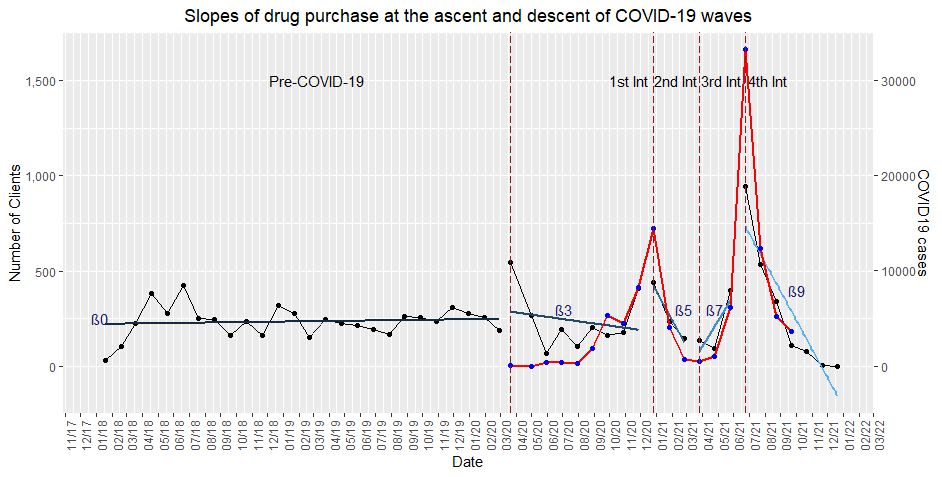

Supplement: S2 Fig — The COVID-19 era is divided into four periods based on the ascent and descent of the two COVID-19 waves in Uganda. The red line shows the monthly number of COVID-19 cases (see secondary vertical axis) reported during the first two waves in the country. The blue line illustrates the slopes and descents estimated from the negative binomial regression model and their corresponding slopes and coefficients. (TIFF) [file pgph.0001579.s002.tiff]
