# Supplementary material for: Community purchases of antimicrobials during the COVID-19 pandemic in Uganda: An increased risk for antimicrobial resistance
Source: PLOS Glob Public Health. 2023 Feb 23;3(2):e0001579. doi: 10.1371/journal.pgph.0001579 (PMC10021632; doi:10.1371/journal.pgph.0001579)
Supplement: Attachment — Submitted filename: Responses to comments 10092022.docx [file pgph.0001579.s004.docx]

**Dear Reviewers,**

I would like to thank the reviewers for the through reviews of the manuscript. The comments are much appreciated and have been addressed accordingly to increase clarity and enrich the manuscript. Please receive a detailed response to each of the comments raised.

**Reviewer 1:**

**Comment 1: The time series model may be adequate, but it is suggested that it better clarify the theory with the results.**

**Response**: To determine the effect of COVID-19 pandemic on customer drug purchases, we employed the single group interrupted time-series analysis (19) using segmented regression.

The number of clients purchasing selected drugs over time (the outcome) were modelled using a negative binomial distribution to account for over dispersion (variance exceeds mean).

Our hypothesis before the study was that the COVID-19 pandemic in Uganda interrupted the time series for drug purchases and created the segments of interest. The observation time was divided into five segments that matched the period before COVID-19 as well as the COVID-19 period, particularly, the ascents and descents of the first and second wave of COVID-19 in Uganda.

The negative binomial regression model generated coefficients(non-transformed) with main outcomes Y being the change in the number of clients purchasing the drug of interest. The coefficients from the negative binomial model were then exponentiated coefficients to obtain ratios that are easy to interpret to and relate to the main outcome of interest. The risk ratios are presented in the results section while model (non-transformed) coefficients are presented in the **Supplementary Table 1.**

**Comment 2: Clarify the specific R package they use.**

**Response:** The following R packages were used: Dplyr package for data management, and the Mass package for Negative binomial regression

**Comment 3: The graphs should be better explained. There are black lines and red lines and you don't know which is which.**

**Response**: The legends for all figures have been edited to provide more details and explanation.

**Comment 4: It seems that the results are not taking advantage of the model, because they are calculating RRs and their confidence intervals, but I think that RRs are not obtained in those models.**

**Response**:

This has been explained in response to comment 2: However, please note that the negative binomial distribution is a generalization of Poisson distribution since it has the same mean structure as Poisson distribution but has an extra parameter to model the over-dispersion in count data. The coefficients (β) are incidence rates whose exponents are incident rate ratios (IRR) or simply Rate Ratios. So, a rate ratio (i.e. exp(coefficient) is the ratio of the rate in group A versus group B).

The negative binomial model takes the same form as the Poisson model. For our case, the model is as below:

log(Yit) = β_0_ + β_1_T + β_2_Z_1_ + β_3_X_1_+ β_4_Z_2_ + β_5_X_2_ + β_6_Z_3_ + β_7_X_3_ + β_8_Z_4_ + β_9_X_4_

which implies

Yit = exp(β_0_ + β_1_T + β_2_Z_1_ + β_3_X_1_+ β_4_Z_2_ + β_5_X_2_ + β_6_Z_3_ + β_7_X_3_ + β_8_Z_4_ + β_9_X_4_)

In the results, we present the IRRs to allow interpretation and comparison of changes in drug purchases during waves. We have added a line for clarity in paragraph of the results section

**Comment 5**

**Figure 1 shows a series with very atypical data that must have an explanation. The unused scale does not show whether this happens for all products or not.**

**Response:** We appreciate the reviewers for highlighting this. This is exactly what we aimed to study and the hypothesis is that drug purchases generally increased during the COVID-19 pandemic. Specifically, the hypothesis is that there were increased drug purchases as COVID-19 cases increased (ascents of the COVID-19 waves) and a decline in purchases as the cases reduced (descents of the waves). In ITSA (interrupted Time Series Analysis) language, the COVID-19 pandemic interrupted the time series of drug purchases with the ascent and descent periods of waves 1 and 2 of COVID-19 pandemic in Uganda leading to five segments in the time series of drug purchases.

Table 1 is from a negative binomial model that has slices at the time points used by the authors, but it is not clear how they get there.

The coefficients (β) are incidence rates whose exponents are incident rate ratios (IRR) or simply Rate Ratios. So, a rate ratio (i.e. exp(coefficient) is the ratio of the rate in group A versus group B).

The model used is as below:

log(Yit) = β_0_ + β_1_T + β_2_Z_1_ + β_3_X_1_+ β_4_Z_2_ + β_5_X_2_ + β_6_Z_3_ + β_7_X_3_ + β_8_Z_4_ + β_9_X_4_

which implies

Yit = exp(β_0_ + β_1_T + β_2_Z_1_ + β_3_X_1_+ β_4_Z_2_ + β_5_X_2_ + β_6_Z_3_ + β_7_X_3_ + β_8_Z_4_ + β_9_X_4_)

Our hypothesis was that drug purchases generally increased during the COVID-19 pandemic. Specifically, the hypothesis was that there were increased drug purchases as COVID-19 cases increased (ascents of the COVID-19 waves) and a decline in purchases as the cases reduced (descents of the waves). In ITSA (interrupted Time Series Analysis) language, the COVID-19 pandemic interrupted the time series of drug purchases with the ascent and descent periods of waves 1 and 2 of COVID-19 pandemic in Uganda leading to five segments in the time series of drug purchases.

**Comment 6: In the final graphs, it seems that they forget the time points.**

**Response**: All graphs have time points on as shown in figures 1 and 2. The time points correspond to the months and years before and after COVID-19. March 2020 corresponds to the month in which the first COVID-19 case was diagnosed. We have added this to the legends for figures 1 and 2 .

**Comment 7: In the last figure they show a pre and post-covid adjustment, it is not clear if they forget the moments of time and why they do it.**

**Response:** The legends for all Figures 1 and 2, and the supplementary figure 1, have been included and more information added for clarity.

**Comment 8: In general, it seems that there is a lack of coherence between the methodological part and the results. I consider that there is a lack of explanation of what is done and what is proposed and how it is explained.**

**Response**: Please see explanation of methods and results in responses to comments 1, 4 and 5. We have explained and presented the original coefficient from the negative binomial model presented in the methodology section, and included the coefficients (non-transformed) in a supplementary table, as well as presented the risk ratios presented in the results section, after exponentiating the regression coefficients.

**Comment 9:**

It is an investigation with an adequate methodology, but with inconsistencies between what they propose and what they present. It could be improved how the results are presented and that the tools used and the reference literature coincide throughout the document.

**Responses:** See explanations in responses to comments 1, 4, 5 and 8

**Comment 10: Bibliography references 16 and 18 are not complete and I consider that they are not necessary.**

Responses: The references have been edited since they refer to the two main data sources for this study – the Vine and Ecopharm pharmacies in Uganda.

**Comment 11: Reference 17 does not seem to correspond to the text.**

Response: Reference has been deleted.

**Comment 12: The abstract uses the term data-mining to do the information set. For me, it is just a collection of data from different sources which they call “supplements” but in results used "micronutrients".**

**Response**: This has been corrected and micro-nutrients have been replaced with supplements throughout the text and the figures.

**Reviewer #2: Dear Authors,**
I have read with interest your manuscript. The purpose of the study, as well as the methods used are relevant. Additionally, using that kind of data opens the door to future studies on sef-medication, which is a field of research very useful to identify key messages to be used in communication campaigns.

**Comment 13:**

Notwithstanding this, I would like to focus os several aspects which I consider important for the present study:
(1) To me, the most relevant point are the selected medicines. I agree that the medicines included should be there. But what about other medicines that were under the spot because of rumours, social media, etc.?
- The most clear example is "Augmentin" (that you already cite as part of a flyer). "Augmentin" should also be included in that analysis.
- But, even more, it would be highly relevant to observe the behaviour of "Augmentin" as compared to the behaviour of other popular brand names of amoxicillin + clavulanic acid. This help to identify the effects of spreading false information using brand names, for example.
- Hydroxychloroquine and chloroquine were also used, especially during the initial stages of the pandemic. The evolution of the sales of both medicines should also be included.
- The same for ivermectin.
- And medicines used to manage fever, such as paracetamol and ibuprofen

**Response:**

We thank the reviewers for pointing the issue of selection of drugs included in the analysis. We chose the selected drugs following trending recommendations of COVID-19 treatment bundles that were disseminated via social media. The bundle included Augmentin– and we have included it in the figures, and the corresponding tables. The issue of brand names and other commonly used analgesics is beyond the scope of this work and the authors will follow-this up in another study. The data requested and provided by the selected pharmacies was restricted to the drugs highlighted on the flyer. We have included a paragraph on limitations of our study with regards to drug selection and we will recommend future studies to identify the effect of COVID-19 on wide range of drug sales.

**Comment 14**

**(3) My last point refers to the limitations of the study, which should be clearly addressed, as well as the differences between the included pharmacies and other pharmacies of the country, sociodemographic aspects, potential different impact of infodemia to clients going to private pharmacies and clients going to public pharmacies, a discussion on the expected behaviour in the other pharmacies, taking into account previous studies, etc.**

**Response**:

In the discussion, the last paragraph has been edited to include the limitation of representation and possible difference between pharmacy sales in urban and rural areas. We have added a reference to a study that showed prices of drugs determine their purchase in resource limited setting.

**Other submission comments**

1. Please amend your detailed online Financial Disclosure statement. This is published with the article. It must therefore be completed in full sentences and contain the exact wording you wish to be published.

a. State the initials, alongside each funding source, of each author to receive each grant.
b. State what role the funders took in the study. If the funders had no role in your study, please state: “The funders had no role in study design, data collection and analysis, decision to publish, or preparation of the manuscript.”

**Response: This has been done – thank you.**

2. Please update your online Competing Interests statement. If you have no competing interests to declare, please state: “The authors have declared that no competing interests exist.”

**Response: This has been done – thank you.**

3. Manuscript datafile for journal.csv is currently uploaded as file type “Other”, which is not viewable by the reviewers. Please change the file type(s) to 'Supporting Information' and include a legend in the manuscript.

**Response: This has been done – thank you.**

4. Please provide separate figure files in .tif or .eps format and remove any figures embedded in your manuscript file. Please also ensure that all files are under our size limit of 10MB.

**Response: This has been done – thank you.**

For more information about how to convert your figure files please see our guidelines: https://journals.plos.org/globalpublichealth/s/figures

5. We notice that your supplementary figure is included in the manuscript file. Please remove them and upload them with the file type 'Supporting Information'. Please ensure that each Supporting Information file has a legend listed in the manuscript after the references list.

Response: This has been done – thank you.
