# Supplementary material for: Community purchases of antimicrobials during the COVID-19 pandemic in Uganda: An increased risk for antimicrobial resistance
Source: PLOS Glob Public Health. 2023 Feb 23;3(2):e0001579. doi: 10.1371/journal.pgph.0001579 (PMC10021632; doi:10.1371/journal.pgph.0001579)
Supplement: Attachment — Submitted filename: Rebuttal letter to PLOS Medicne.docx [file pgph.0001579.s005.docx]

**PLOS Global Public Health Decision**

**Article number: PGPH-D-22-00781R1 - [EMID:6ac198e9bf272a6c]**

**Rebuttal letter to Editors’ comments**

**Comment: Uploading** Manuscript data file for journal.csv.

*Response*: The file has been uploaded as “supporting information and referenced in the manuscript.

**Comment: Language and grammar check:**

Response: The manuscript has been reviewed and all grammatical errors have been corrected.

Reviewer #3 comments:

**Comment: Title is too long.**

**Response:** The title has been shortened to read as shown below; “Community Purchases of Antimicrobials during the COVID-19 Pandemic in Uganda: An increased risk for antimicrobial resistance

**Comment: Methodology: No clear design.**

*Response*: This has been edited and included in the first paragraph of the methods section.

**Comment: I think it would be more useful if you include regression model explanation in your Methodology section of the manuscript instead of writing it in the Supplementary figure file. This would be easier for the readers and provide more clarity on your work.**

*Response*: The information on the model has been inserted in the methods section.

**Comment: The legend for data used is still not present in the manuscript. Please follow PLOS convention for uploading data as Supplementary Information.**

*Response*: The manuscript datafile has been uploaded as supplementary information, and referenced in the methods section.

**Comment: Table 1 has inconsistencies in representation of Confidence intervals. Please adjust the parentheses.**

*Response***:** The table has been corrected and parentheses are edited accordingly.

**Comment: Figure 1 and 2 titles are confusing. Please re-write, for example, if the graphs are from interrupted time series analysis results or actual purchases.**

*Response*: Figure 1 has been renamed as a supplementary figure 1, as the same information is presented in Figure 2. The figure legends have been edited for clarity.

**Comment: You could include Strengths and Limitations with a different heading under Discussion section.**

*Response*: This has been included in the discussion.

Reviewer #5:

**Comments on Abstract
Line number 3, ‘’aimed to’’ deleted, and ‘’d’’ added to estimate
Recommendations was not included in the abstract. This should be included.
Keywords should be included**
*Response*: The grammatical errors have been corrected. Recommendations and key words have been added.

**Comments: Introduction section**

**Line number 7 to be deleted. It is repetition of Line number 6.
Line number 8 (‘’Previous studies have estimated the prevalence………….) to be merged with Line number 6
Line number 15 (Need for authors to provided reference for the sentence: ‘’In Uganda, the second wave of the SARS-CoV2 Delta Variant…………by April 25, 2022
Line number 17: Need for authors to include Reference to the ending of the statement from Line number 16 (……., including antibiotics to treat symptoms of COVID-19 disease)**
*Responses*: All the above edits have been made in the revised manuscript.

**Comment: Need for authors to clearly reframe the objectives of the manuscript**
*Response:* A line has been included in the last paragraph of the introduction section, to highlight the study objective.

**Comment: Change Methodology to Methods**

*Response*: This has been corrected.

**Comment: Information under Data variables will be better presented using a flow diagram**

*Response*: Thank you for the suggestion, however we feel that the current presentation of the variables is sufficient, give that there were not many exclusions in the data selection, to warrant a fully informative flow diagram.

**Comment: Need for operational definitions of some terms under the methods section: prescribed, non-prescribed, pre and post COVID-19 pandemic periods etc**

**Response:** Thank you for the comment. The definitions for each of the key terms are included in the text for clarity.

**Comment: ‘’To determine the effect of COVID-19 pandemic on customer drug purchases’’ appears to be part of the study objectives which supposed to be well articulated under the Introduction section**

*Response*: Thank you for the comment. We have included this sentence under the introduction. However, we have similarly edited the very same sentence as it provides context for using the interrupted time-series analysis presented in the methods section.

**Comment: ‘’Our hypothesis before the study was that……..created the segments of interest’’ supposed to be under the Introduction section**

*Response*: The sentence was redundant and has been removed.

**Comment: Data analysis section to have sub-sections for easy and well articulate flow of information**

*Response*: We have added a section for the model with easier interpretation of the equations used in the regressions. All the other sections are clear and easy to flow.

**Comments: Results
Line number 1 ‘’During the pre-Covid era 11th March 2020’’. This should be captured under the Methods section**

Response: This has been captured in the methodology section and only presented in parenthesis to remind the reader of the study period.

**Comment: Supplements: Table 1 to be included at the end of the sentence under supplements. Corticosteroids: Table 1 to be included at the end of the sentence under corticosteroids**

*Response*: The changes have been effected.

**Comment: Discussion
Line number 35: ‘’the role of’’ to be deleted from ‘’It therefore clear that…….’’**

*Response*: This has been corrected.

**Comment: Tables
Incomplete information provided as title. Need to include information on where and when**

**Figures
No title. Need to insert complete title that will provide information on ‘’What, Where, and When’’**
*Response*: The titles of the table and the figure legends have been edited. Thank you.

**Comment: References
URL to be provided with date accessed most especially for references from WHO website
For references with URL being provided, authors should include date when the information was assessed**

*Response*: All the references have been reviewed and corrected.
